# Inhibitors of Immune Checkpoints: Small Molecule- and Peptide-Based Approaches

**DOI:** 10.3390/jpm14010068

**Published:** 2024-01-04

**Authors:** Natalie Fuchs, Longfei Zhang, Laura Calvo-Barreiro, Katarzyna Kuncewicz, Moustafa Gabr

**Affiliations:** 1 Molecular Imaging Innovations Institute (MI3), Department of Radiology, Weill Cornell Medicine, New York, NY 10065, USA; nsf4003@med.cornell.edu (N.F.); loz4003@med.cornell.edu (L.Z.); lac4018@med.cornell.edu (L.C.-B.); katarzyna.kuncewicz@ug.edu.pl (K.K.); 2 Faculty of Chemistry, University of Gdańsk, 80-308 Gdańsk, Poland

**Keywords:** immunotherapy, drug discovery, small molecules, immunomodulators, cancer therapeutics, peptides, nanomaterials

## Abstract

The revolutionary progress in cancer immunotherapy, particularly the advent of immune checkpoint inhibitors, marks a significant milestone in the fight against malignancies. However, the majority of clinically employed immune checkpoint inhibitors are monoclonal antibodies (mAbs) with several limitations, such as poor oral bioavailability and immune-related adverse effects (irAEs). Another major limitation is the restriction of the efficacy of mAbs to a subset of cancer patients, which triggered extensive research efforts to identify alternative approaches in targeting immune checkpoints aiming to overcome the restricted efficacy of mAbs. This comprehensive review aims to explore the cutting-edge developments in targeting immune checkpoints, focusing on both small molecule- and peptide-based approaches. By delving into drug discovery platforms, we provide insights into the diverse strategies employed to identify and optimize small molecules and peptides as inhibitors of immune checkpoints. In addition, we discuss recent advances in nanomaterials as drug carriers, providing a basis for the development of small molecule- and peptide-based platforms for cancer immunotherapy. Ongoing research focused on the discovery of small molecules and peptide-inspired agents targeting immune checkpoints paves the way for developing orally bioavailable agents as the next-generation cancer immunotherapies.

## 1. Introduction

The landscape of cancer treatment underwent a paradigm shift with the inception of immunotherapy, tapping into the body’s innate defense mechanisms against cancer cells [1,2,3]. Within this context, immune checkpoints, exemplified by programmed cell death protein 1 (PD-1) and cytotoxic T-lymphocyte-associated protein 4 (CLTA-4), have emerged as pivotal orchestrators of immune function [4,5,6]. Tumors have the ability to evade immune surveillance through the utilization of immune-escape mechanisms, which involve creating an immunosuppressive microenvironment and inhibiting the function of effector T cells within the tumor microenvironment [7,8]. The goal of cancer immunotherapy is to rekindle the anti-tumor immune response, intensifying its effects to counteract tumor-induced immune suppression [9,10,11]. One of the most efficacious approaches involves activating T cell-mediated anti-tumor responses, primarily through the modulation of immune checkpoints. These checkpoints are pivotal receptors that play crucial roles in preventing autoimmunity, safeguarding the host from tissue damage, and regulating self-tolerance [12,13,14]. The activation of T cells specific to cancer plays a pivotal role in eradicating cancer cells through the recognition of tumor-specific antigens [15,16]. Initially, antigens are presented by antigen-presenting cells (APCs) in the form of antigenic peptides, which are identified by the T cell receptor [17]. Subsequently, B7 proteins (CD80 and CD86) on the APCs interact with CD28 on T cells, resulting in T cell activation [18]. Following activation, cancer-specific T cells migrate to the tumor sites, where they identify and eliminate cancer cells by recognizing tumor-specific antigens [18]. However, the tumor microenvironment poses challenges, as cancer cells often exhibit an elevated expression of co-inhibitory protein ligands, including CD80/86 and programmed death-ligand 1 (PD-L1) [19,20,21]. Co-inhibitory proteins, such as CTLA-4 and PD-1, bind to their corresponding ligands on cancer cells, resulting in the prevention of cancer-specific T cell activation and the escape of cancer cells from immune surveillance [22,23,24]. Thus, the inhibition of the interaction between negative immune checkpoints and their binding partners has been extensively pursued as an effective platform for cancer immunotherapy.

Currently, multiple monoclonal antibodies (mAbs) are approved by the U.S. Food and Drug Administration (FDA) as immune checkpoint inhibitors for various malignancies [25]. Although these mAbs have revealed remarkable clinical success for a subset of cancer patients, limitations such as immune-related adverse effects (irAEs), immunogenicity concerns, and elevated costs represent constraints for the clinical utility of mAbs as immune checkpoint inhibitors [26,27,28,29,30,31]. The manifestation of irAEs by mAbs may be influenced by sustained target inhibition due to an extended half-life (>15–20 days) and a target occupancy exceeding 70% for prolonged periods [32,33]. Unlike mAbs, peptides and small molecules possess smaller molecular weights, reduced immunogenicity, improved tissue and tumor penetration, and lower manufacturing costs [34,35,36,37,38]. Notably, small molecules lend themselves more readily to pharmacokinetic optimization, enabling the adoption of flexible dosage regimens that could help avoid irAEs associated with mAbs. The assessment of the allergenic potential of small molecules and peptides involves a combination of experimental and computational methods, such as (1) in silico analysis (e.g., simulations of the interactions with immune cell receptors); (2) in vitro experiments (e.g., basophil activation test (BAT); (3) in vivo models for allergenic potential; and (4) epidemiological data. Peptides and small molecules hold significant promise as complements to mAb-based therapy, offering the potential for enhanced synergistic effects. The advantages of incorporating small molecules and peptides in cancer immunotherapies would be highly remarkable as the field progresses toward synergistic combination therapies designed to target multiple receptors and aiming to amplify the overall response rates of cancer immunotherapy approaches. In this review, we seek to investigate the latest advancements in the targeting of immune checkpoints, with a specific focus on both small molecules and peptide-based methods. By examining various drug discovery platforms, we aim to offer insights into the wide range of strategies utilized for the identification and optimization of small molecules and peptides as inhibitors of immune checkpoints.

## 2. Small Molecules as Immune Checkpoint Inhibitors

### 2.1. Random and Focused Screening Approaches

**Figure 1:**
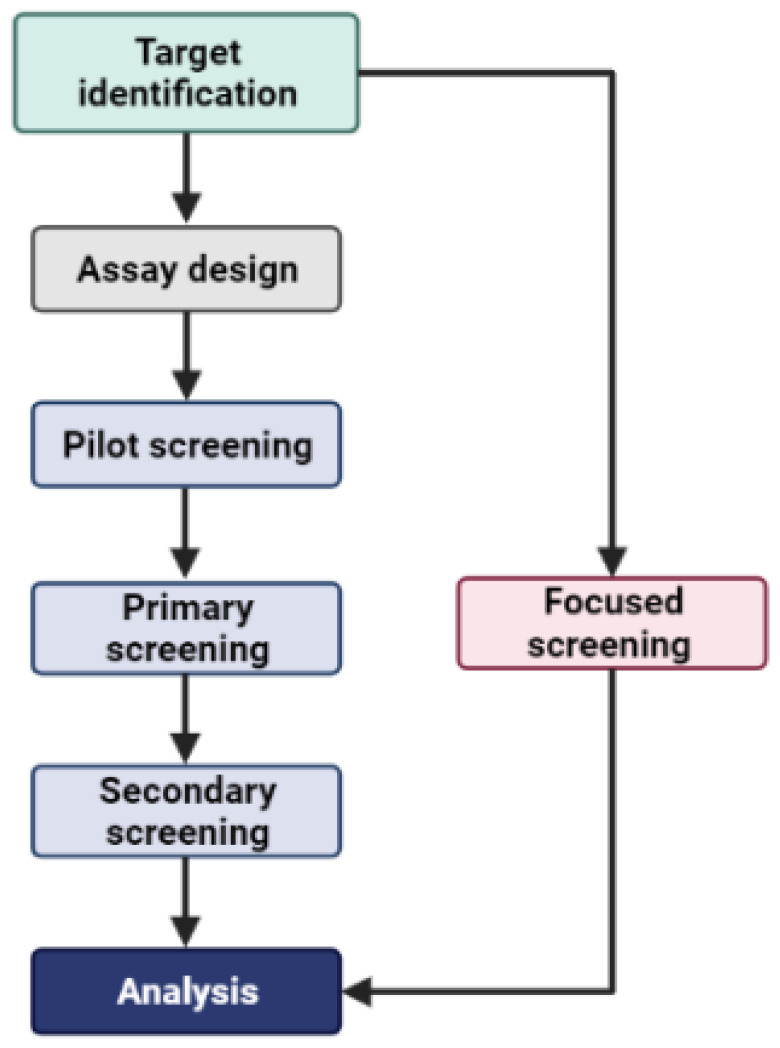
Demonstration of the key difference between random screening and focused screening approaches. Focused screening enables bypassing of the multiple stages of screening associated with random screening.

High-throughput screening (HTS) involves screening extensive chemical libraries of small molecules in three phases: pilot, primary, and secondary (Figure 1). In the primary phase, where approximately 200 k compounds are screened on average, using HTS is akin to searching for a needle in a haystack [39,40,41]. The initial selection of the search area significantly influences the success rate. Two major challenges in screening are identifying the right compounds and covering a broad chemical space to comprehend their biological function [39,40,41]. Alternatively, employing computational methods to identify ligand binding sites in proteins allows the use of virtual screening algorithms [42,43,44,45] to create a focused library of chemical compounds (Figure 1). Developing focused chemical libraries through virtual screening can streamline the drug discovery process, optimizing screening and yielding better results while reducing costs. Additionally, this approach enhances the diversification of relevant scaffolds for further hit-to-lead optimization efforts.

**Figure 2:**
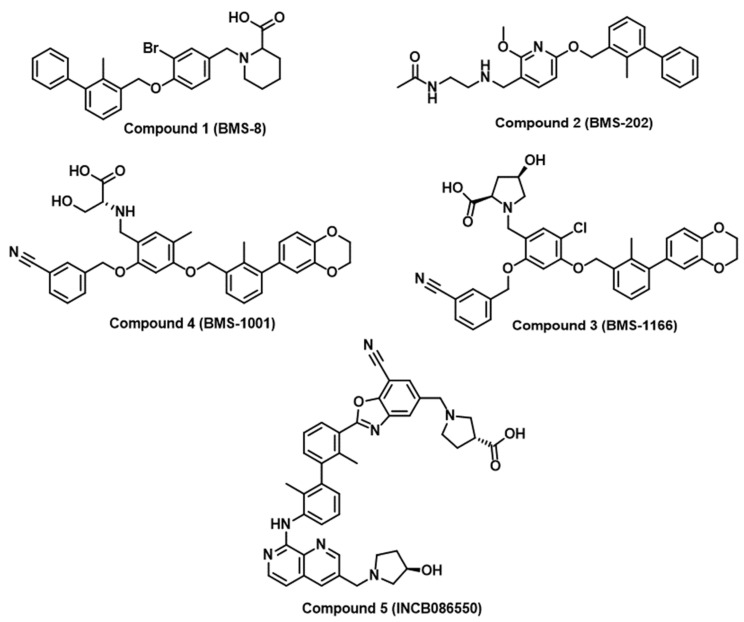
Chemical structures of reported small molecule PD-L1/PD-1 inhibitors from BMS and Incyte Corporation.

There are few successful examples of the implementation of rational medicinal chemistry approaches for the discovery of small molecule inhibitors of immune checkpoints. Researchers at Bristol Myers Squibb (BMS) have disclosed a set of substituted biphenyl derivatives, highlighting their efficacy in inhibiting the interaction between PD-1 and PD-L1 [36]. Representative examples from the BMS compounds are included in Figure 2 (Compounds **1**–**4**). Nevertheless, there is no available information on the progression of these intriguing yet notably hydrophobic small molecules into clinical applications. Numerous companies, such as Incyte Corporation, Arising International Inc., Chemocentryx Inc., Polaris Pharmaceuticals, and Guangzhou Maxinovel Pharmaceuticals Co., have identified a range of small molecule PD-L1 inhibitors utilizing the biphenyl core [29,36]. The most successful outcome in this context is the development of INCB086550 (Compound **5**, Figure 2) with a demonstrated reduction in tumor growth in humanized mice with CD34^+^ cells and elicited gene signatures associated with T cell activation, aligning with the blockade of the PD-L1/PD-1 pathway [46]. Early findings from an ongoing phase I study affirmed the blockade of PD-L1/PD-1 in peripheral blood cells, showing heightened immune activation and effective control of tumor growth, providing a basis for a further clinical assessment of INCB086550 as a potential alternative to antibody-based therapies [46]. Notably, many academic groups have attempted to optimize BMS compounds as PD-L1/PD-1 inhibitors [47,48,49,50,51,52]. However, the scarcity of validated hits as PD-1 inhibitors and the restriction to PD-1 inhibition have directed research efforts towards virtual screening with the aim of identifying small molecule inhibitors of PD-1 and other immune checkpoints.

**Figure 3:**
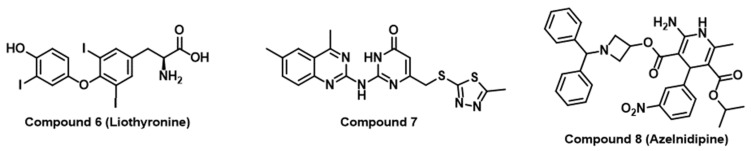
Chemical structures of small molecule immune checkpoint inhibitors identified using virtual screening approaches.

Numerous reports have validated virtual screening as a successful approach to identifying novel small molecule PD-L1/PD-1 inhibitors [53,54,55]. Importantly, the employment of computational approaches has enabled the expansion of small molecule drug discovery efforts to various immune checkpoints other than PD-1 [56,57,58]. For example, the molecular docking of a focused chemical library to poliovirus receptor PVR (also known as CD155 and Nectin like-5) using the Molecular Operating Environment (MOE) software (version: 2016.08) resulted in the discovery of liothyronine (Compound **6** in Figure 3) as a PVR binder and an inhibitor of the interaction between PVR and T cell immunoglobulin and ITIM domain (TIGIT) [56]. Liothyronine has revealed the ability to augment the activity of CD4^+^ and CD8^+^ T cells in peripheral blood mononuclear cells (PBMCs) [56]. Additionally, in a coculture assay involving Jurkat-hTIGIT and CHOK1-hPVR, liothyronine demonstrated the ability to reverse the inhibition of IL-2 secretion caused by TIGIT/PVR ligation [56]. Remarkably, liothyronine significantly impeded tumor growth when administered in vivo by enhancing CD8^+^ T cell infiltration and immune responses in tumor-bearing mice [56]. In addition, homology modeling of the 3D structure of the V-domain Ig Suppressor of T cell activation (VISTA) and subsequent virtual screening resulted in the identification of Compound **7** (Figure 3) as a VISTA binder with submicromolar VISTA binding affinity and potent immunomodulatory activity in coculture cellular assays [57]. Another successful example of the implementation of molecular docking studies using MOE is represented by the discovery of Azelnidipine (Compound **8**, Figure 3) as a dual inhibitor of TIGIT/PVR and CD47/SIRPα along with the demonstration of the significant inhibition of the growth of CT26 tumors in vivo by Azelnidipine based on enhancing the infiltration and function of the CD8^+^ T cell in the tumor [58]. 

**Figure 4:**
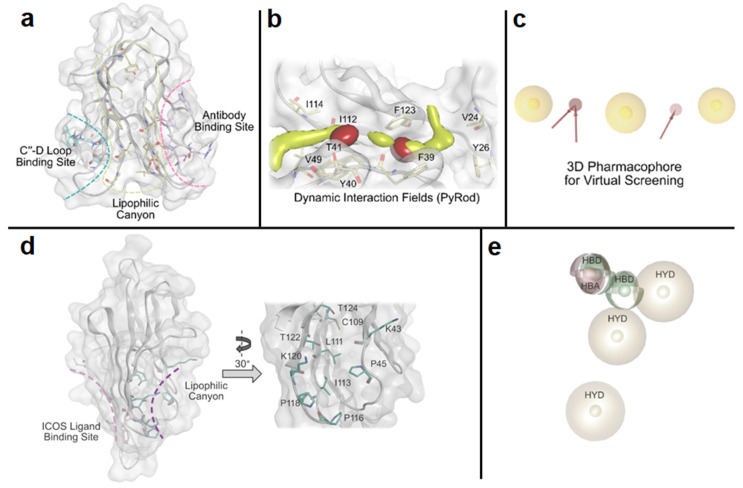
(**a**) Global view on TIM-3 showing the previously targeted binding pockets and a novel binding site for small molecules (lipophilic canyon) [59]. (**b**) Dynamic molecular interaction fields (dMIFs) obtained from PyRod. Color code: yellow clouds—lipophilic areas; red clouds—hydrogen bond accepting areas [59]. (**c**) Three-dimensional pharmacophore model derived from the dMIFs used for the virtual screening campaign to identify small molecule TIM-3 binders [59]. (**d**) Global view of the ICOS structure, ICOS-L binding site (pink), and lipophilic canyon (purple), along with a zoom into the lipophilic canyon analyzed using PyRod [60]. (**e**) Three-dimensional pharmacophore used for virtual screening after dMIF analysis to identify small molecule ICOS binders [60]. Color code: yellow spheres: hydrophobic contacts (HYD); red spheres: hydrogen bond acceptor (HBA); green spheres: hydrogen bond donor (HBD).

Our laboratory has pioneered the discovery of first-in-class small molecule inhibitors of immune checkpoints using random, focused, and computational-based screening approaches [59,60,61,62,63,64]. In the context of virtual screening, we recently reported the implementation of a pharmacophore-based virtual screening approach to identify small molecule inhibitors of T cell immunoglobulin and mucin domain 3 (TIM-3) [59]. We discovered a potential lipophilic binding pocket with a canyon-like topology of TIM-3 (PDB ID: 7M3Z, Figure 4a). To analyze the lipophilic canyon of TIM-3, we performed molecular dynamics (MD) simulations in order to apply PyRod, a tool that analyzes the trajectories of MD simulations and automatically generates a set of dynamic molecular interaction fields (dMIFs, Figure 4b) derived from solvent interactions. We used these dMIFs to develop a 3D pharmacophore model (Figure 4c) located in the lipophilic canyon of TIM-3. Virtual screening based on this pharmacophore model resulted in the identification of a small molecule binder of TIM-3 with submicromolar affinity and the ability to modulate TIM-3/ligands interactions [59]. Analogously, we conducted a computational study that resulted in the discovery of first-in-class small molecule binders of inducible co-stimulator (ICOS), an activating costimulatory immune checkpoint expressed on activated T cells [60]. We detected a lipophilic canyon adjacent to the binding site of the physiological ligand of ICOS (ICOS-L), presenting a potential binding site for small molecules (Figure 4d). Subsequently, we employed PyRod to generate a 3D pharmacophore (Figure 4e) within the identified lipophilic canyon, which was utilized for virtual screening [60]. Notably, we validated and identified the ICOS binding affinity of the identified hits using both microscale thermophoresis (MST) and surface plasmon resonance (SPR) screening [60].

### 2.2. Immune Checkpoint-Targeting Degraders and Covalent Inhibitors

Since most immune checkpoints are designed for protein–protein interactions (PPIs), developing small molecule-based inhibitors remains challenging [65]. Most immune checkpoints’ flat hydrophobic binding pockets limit interactions with small molecule ligands, resulting in reduced efficacy [65]. As detailed above, several efforts have been made to develop small molecule-based non-covalent checkpoint inhibitors, most prominently the BMS compounds and their derivatives [36,37,38,39,40,41,42,43,44,45,46,47,48,49,50,51]. However, as of today, no small molecule-based checkpoint inhibitors have received approval from drug admission boards. Considering the crucial role of residence time in inhibition efficacy, enhancing an inhibitor’s efficiency can be achieved by introducing a mildly reactive group for covalent binding to the target [66,67,68]. Designing small molecule-based covalent inhibitors relies on rational reactivity and selectivity fine-tuning [69,70]. Targeted covalent inhibition has proven to be effective across various proteins, leading to the development of several FDA-approved drugs over the past two decades. Examples include protease inhibitors for viral infections (e.g., nirmatrelvir) and myeloma treatment (e.g., bortezomib) or kinase inhibitors for cancer therapy (e.g., afatinib) [71,72,73,74,75].

**Figure 5:**
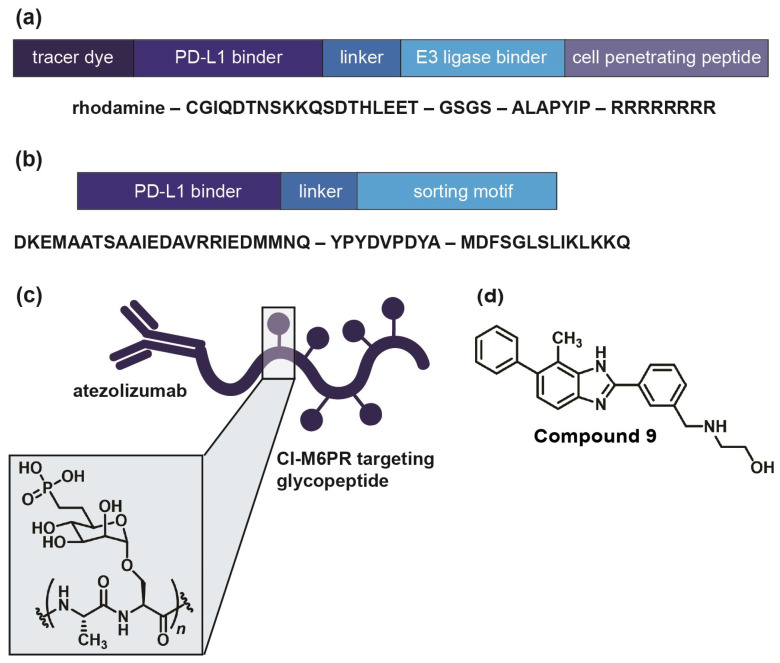
Examples of immune checkpoint-targeting degraders. (**a**) PD-L1-targeting PROTAC, as published by Dai et al. [82]. Their peptide consists of a PD-L1 binding sequence linked to an E3 ligase binder with a C-terminal cell penetrating Arg sequence. (**b**) PD-LYSO, as developed by Wang et al. [90]. A PD-L1 binding peptide is linked to a lysosomal sorting motif that facilitates binding to HIP1R. (**c**) PD-L1 targeting LYTAC, as published by Banik et al. [84]. Atezolizumab is linked to a CI-M6PR targeting glycopeptide with poly-mannose-6-phosphate. (**d**) Dual VISTA inhibitor and degrader, as developed by Wang et al. [99]. Their compound triggers selective autophagy of the target protein through elevated levels of autophagosome marker LC3-II.

In the field of immune checkpoints, efforts to develop covalent inhibitors have been limited so far. Li and co-workers have proposed a new approach called proximity-enabled reactive therapeutics (PERx) that involves incorporating unnatural amino acids into proteins, such as the bioreactive fluorosulfate-l-tyrosine (FSY) [76]. FSY selectively reacts with a proximal histidine in the target protein PD-L1, resulting in covalent irreversible binding. They were able to demonstrate the anti-tumor effect of PERx in vitro and in vivo with an efficacy that is comparable to or even surpassing that of anti-PD-L1 antibodies [76]. This underscores the viability of covalent inhibition as a strategy to target immune checkpoints, urging further exploration, including with small molecules. If covalent inhibition of the target protein is not feasible, an alternative approach involves inducing the degradation of the immune checkpoint. There are several strategies for targeted protein degradation, starting out with proteolysis-targeting chimera (PROTAC) [77,78,79]. This is a chemical knockdown method using heterobifunctional conjugates. One part of the construct binds to the target protein, whereas the other part recruits an E3 ubiquitin ligase, leading to the ubiquitination of the target protein. Once ubiquitinated, the protein of interest will be degraded by the proteasome [77,78]. Engaging a physiological enzyme cascade has proven to be a potent, selective, and reversible alternative to RNAi and CRISPR knockdown both in vitro and in vivo. Numerous investigations have explored PROTACs to target PD-L1 (e.g., Figure 5a), including antibody-based PROTACs (AbTACs) consisting of recombinant bispecific antibodies or peptide-based PROTACs [80,81,82]. Nevertheless, small molecule-based approaches also exist, as exemplified by Cheng and co-workers [83]. They developed resorcinol diphenyl ether-based PROTAC-like small molecules that are both PD-L1 inhibitors and degraders. Their lead compound, **P22**, was able to restore the immune response in a T cell tumor co-culture model and to moderately degrade PD-L1. However, their findings suggest that **P22** mediates a lysosomal degradation pathway for PD-L1 rather than the intended proteasomal pathway [83]. 

Generally, recruiting an E3 ubiquitin ligase and using the proteasomal pathway is conventionally confined to cytosolic protein domains [84]. Therefore, lysosomal degradation represents a more suitable approach for membrane-associated proteins like immune checkpoints [84,85,86]. Lysosomes facilitate intracellular protein degradation via three different pathways: endocytosis, phagocytosis, or autophagy [85,87,88]. Endocytosis requires a target binder conjugated with a lysosomal sorting motif, for example, the di-leucine motif [86,89]. This motif can initiate protein degradation upon binding to the adaptor protein (AP) complex, inducing checkpoint endocytosis and import to the multivesicular body (MVB) of the lysosome [90]. Wang and co-workers discovered a PD-L1-binding peptide based on functional motifs of the Huntingtin Interacting Protein 1 (HIP1R). They incorporated this PD-L1 binding sequence and the lysosomal sorting motif into one peptide (PD-LYSO, Figure 5b) and observed a successful decreased PD-L1 expression in tumor cells [90]. Banik and co-workers took it even further and introduced the concept of “lysosome-targeting chimera”, LYTAC in short, which is suitable for membrane-associated proteins as well as extracellular proteins [84]. LYTACs comprise conjugates that bind to both the target protein’s extracellular domain and a lysosome-shuttling receptor, such as the cation-independent mannose-6-phosphate receptor (CI-M6PR) [91,92]. They designed a LYTAC consisting of anti-PD-L1 (atezolizumab) conjugated to glycopeptide ligands as CI-M6PR agonists (Figure 5c), resulting in 50–70% degradation in MDA-MB-231 cells after 36 h of treatment [84]. Most recently, Li et al. engineered a chimeric DNA aptamer targeting CI-M6PR that allows for PD-L1 binding through click reactivity on the tumor cell membrane [93]. In the future, the LYTAC approach could be applied to small molecule ligands as well. Selective autophagy is another option to mediate lysosomal protein degradation since it regulates many immune checkpoints [88,94,95]. One possibility is chaperone-mediated autophagy [96]. Here, a sorting motif, e.g., the KFERQ peptide, triggers the regulated delivery of cytoplasmic components to the lysosome [97,98]. This motif is then conjugated to a cell membrane-penetrating domain as well as a target protein-binding domain. To date, the KFERQ peptide has not yet been successfully incorporated for checkpoint degradation. Nevertheless, Wang and co-workers demonstrated how to use the autophagy pathway to degrade the immune checkpoint VISTA [99]. They designed small molecules with a benzimidazole core as bifunctional VISTA inhibitors with binding affinity in the submicromolar range. Their lead (Compound **9**, Figure 5d) has been proven to promote VISTA degradation while increasing the expression of lipidated MAP1LC3 (LC3-II), an autophagosome membrane marker in HepG2 cells [100]. Their findings suggest an autophagy-dependent VISTA degradation caused by Compound **9**, which they verified with a cycloheximide chase assay as well as with Western blots. Compound **9** was also active in a CT26 mouse model, significantly suppressing tumor growth [99]. 

**Figure 6:**
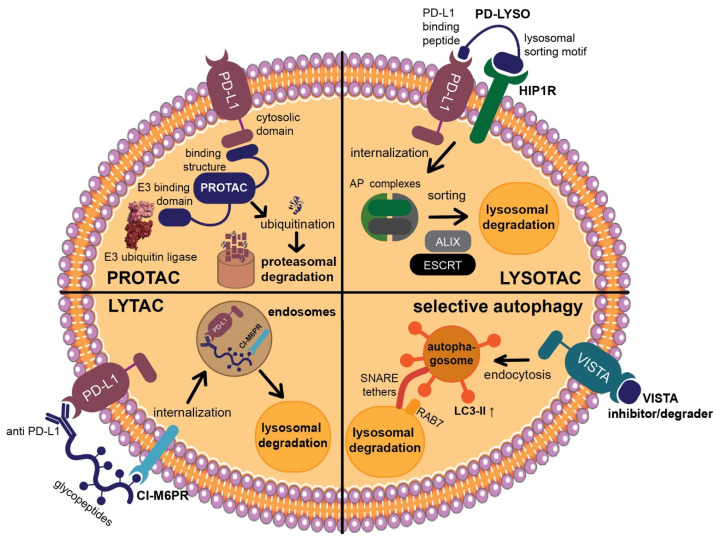
Overview of protein degradation pathways suitable for immune checkpoints. **PROTAC** (**top left**): Binding of its cytosolic domain as well as an E3 ligase results in ubiquitination and proteasomal degradation of PD-L1 [78,81,82]. **LYSOTAC** (**top right**): Combining a PD-L1 binding peptide with a lysosomal sorting motif leads to internalization and lysosomal degradation of the target protein [90]. **LYTAC** (**bottom left**): An anti-PD-L1 antibody coupled to a glycopeptide sequence can address PD-L1 and CI-M6PR, resulting in internalization and lysosomal degradation of PD-L1 [84,102]. **Selective autophagy** (**bottom right**): A dual VISTA inhibitor/degrader raises expression levels of autophagosome marker LC3-II, leading to increased VISTA degradation in the lysosome through autophagosomal endocytosis [99,103,104].

In conclusion, there are several strategies regarding protein degradation that can be suitable for immune checkpoints (Figure 6). The efficacy of PROTACs or LY(SO)TACs is not limited by the target’s equilibrium occupancy, as is the case for traditional inhibitors. Therefore, degraders are active in a catalytical manner, allowing them to be efficient at low concentrations [101]. While degraders must still be good binders for their target proteins, they do not have to be intrinsically biologically active as inhibitors [101]. These advantages make targeted protein degradation an attractive concept that can be explored with small molecules, and might also expand the range of druggable proteins, particularly immune checkpoints.

### 2.3. DNA-Encoded Library Screening as a Powerful High-Throughput Technology in Drug Discovery

DNA-encoded libraries (DELs) are collections of small molecule compounds that are covalently linked to unique DNA tags, which act as molecular barcodes and allow for compound identification [105]. Sydney Brenner and Richard Lerner are often credited as the pioneers in DEL technology development when exploring the idea of encoding individual members of a large library of chemicals with unique nucleotide sequences [106]. Although the chemistry behind the design and synthesis of these libraries is diverse and has evolved over the past three decades [107], the most common approach is the split and pool DNA-encoded synthesis [108]. Briefly, the synthesis starts with an oligonucleotide containing a chemical linker moiety that will be elongated through parallel synthesis and encoding with different chemical building blocks and DNA codes, respectively. As a result, just as many different compounds as building blocks that are added to the reaction will be obtained. After, all compounds are pooled and split again to perform as many cycles of parallel encoding and synthesis as desired. Finally, after two to four repetitive cycles of elongating the DNA barcode and conducting chemical synthesis, libraries containing millions to billions of compounds are often created. 

One of the main advantages of using DNA tags to identify chemical compounds is to allow the high-throughput screening of large small-molecule libraries and perform a precise identification of the binders. Following the binding of the small molecule to the target, the DNA tag is amplified by DNA replication and, ultimately, DNA sequencing is employed to decode the chemical structure. Thus, the DEL screening approach allows for the identification of potential drug candidates in an efficient and cost-effective manner [109]. Likewise, DEL technology has increased the size and diversity of current repository compounds that were previously surpassed by the advancements in laboratory screening capacity, which can now handle more than 100,000 compounds per day [110]. 

Besides the description of successful approaches on cell membrane proteins, such as the insulin receptor [111], the tumor-associated membrane protein, CAIX (carbonic anhydrase IX) [112], and immune receptor, NKG2D (natural-killer group 2, member D) [113], G protein-coupled receptors (GPCRs) have widely been the target of DEL screening research. These receptors play crucial roles in numerous cellular processes [114], immune-mediated diseases [115], and cancer [116,117]. The β2-adrenergic receptor (β2AR), part of the GPCR family, is mainly expressed in pulmonary and cardiac muscles and has been targeted for the treatment of cardiovascular and respiratory diseases, such as asthma, with antagonist and agonist drugs, respectively [118]. A 190-million small molecule DEL was screened for the β2AR to find an allosteric antagonist, which does not compete with β2AR orthosteric ligands [119]. Remarkably, this small molecule, which displays a low micromolar affinity to β2AR, halts the binding of agonistic compounds to the receptor, enhancing its inactive state [119]. On the other hand, the screening of a 500-million DEL on β2AR bound to its high-affinity agonist, BI-167107, led to the discovery of a small molecule compound with positive allosteric modulation properties [120]. In contrast to the β2AR allosteric antagonist, this agonistic allosteric modulator shows cooperation with other β2AR orthosteric agonists, enhancing their binding and stabilizing β2AR active states [120]. Different research developed a focused DEL targeting angiotensin II type I receptor (AT1R) and endothelin receptor A (ETAR), which are GPCRs connected to inflammation and vascular functions [121,122]. This 32,000-compound library was designed based on the use of phenolic acids as building blocks due to their previously described therapeutic cardiovascular applications [121,122]. While the immobilized ETAR-affinity chromatography approach ended up with the discovery of two drug candidates with some potential to become leads for subsequent investigation [122], the screening of the same library using the immobilized AT1R-based chromatographic technique resulted in the detection of a hit with a high picomolar range affinity to the target receptor [121]. Subsequent in vivo studies in renovascular hypertensive rats (two-kidney two clip method) demonstrated that the identified AT1R hit at a dose equal to or higher than 15 mg/kg body weight performed an antihypertensive activity, reducing both systolic and diastolic blood pressure [121].

Regarding immune checkpoints, there is only one research study to our knowledge that has described the discovery of a small molecule targeting TIGIT [123]. This small molecule is capable of inhibiting TIGIT interaction with one of its natural ligands, CD155 [123]. This novel immune checkpoint target has been described as a promising therapy for cancer treatment [124,125], and more than 50 clinical trials are currently recruiting patients or being conducted that involve the use of anti-TIGIT therapies alone or in combination with other immune checkpoint inhibitors, such as PD-1 or PD-L1 (https://clinicaltrials.gov/, accessed on 22 November 2023). Although no information is detailed on the rationale behind the construction of the 30-million compound library, the success on finding one TIGIT/CD155 complex inhibitor after affinity binding screening with the TIGIT protein alone might be related to the study of the complex hot spot before library construction. After, 34 different small molecule derivatives were synthesized, and their TIGIT/CD155 inhibiting capacities were evaluated. A total of 7 out of the 34 derivatives presented improved IC_50_ values compared to the original hit (IC_50_ = 20.7 µM), and both their structures and IC_50_ values were used in the construction of a machine learning model [123]. As a result, information on the molecule fragments that are key to understanding the structure–activity relationship as well as to improve target affinity were obtained. However, although this approach might help the establishment of later models, the small sample size to train the model kept its performance low [123].

Besides the in vitro HTS for small molecule drug discovery, the use of DNA-encoded libraries in mammalian cells is being developed as a promising avenue for drug discovery [126,127,128]. This innovative and evolving field would allow for the study of small molecule interactions with their biological target within the complex cellular environment, which is frequently needed to keep both the structure and functionality of target proteins. One of these developed strategies is a new system based on bioluminescence resonance energy transfer (BRET) [129]. Briefly, BRET is a proximity-based assay like the widely known FRET (fluorescence resonance energy transfer) that does not require a laser as the external light source to excite the donor. On the contrary, the use of a luciferase (NanoLuc) tagged to the target protein works as the donor component that excites a cell-permeable fluorescent probe introduced into live cells [130]. If the equilibrium between the luciferase-tagged target protein and the fluorescent probe is halted by the binding of an unmodified small molecule, a loss of the BRET signal in live cells will be observed. Thus, this (and other) platforms would be of great interest to the cellular validation of previously obtained hits and to speed up the hit-to-lead research.

Along with academic research, industry has also focused its resources on clinical translational opportunities. Such is the case with the Confo Therapeutics and DyNAbind drug discovery collaboration on the investigation of DEL for the discovery of novel small molecules with the capacity of GPCR modulation (https://www.confotherapeutics.com/2019/07/03/confo-therapeutics-and-dynabind-announce-drug-discovery-collaboration-to-identify-novel-gpcr-modulating-compounds/, accessed on 22 November 2023). Additional pharmaceutical companies have also developed and invested in DEL technology, such as GSK [131,132], AbbVie (https://www.abbvie.com/science/areas-of-innovation/advanced-technologies.html, accessed on 22 November 2023) and Amgen (https://www.amgen.com/stories/2019/11/dna-encoded-libraries-will-drive-new-drug-design-paradigm, accessed on 22 November 2023), among others, by developing their own libraries and technologies to target and screen their targets of interest.

## 3. Peptides as Immune Checkpoint Inhibitors

Peptides are gaining increasing attention from researchers, as evidenced not only by the increasing number of publications on immune checkpoint peptide inhibitors, but also by newer methods that allow for the faster development of immune checkpoint-targeting peptides and their appropriate formulation. One of the most widely used methods is rational peptide design based on protein–protein interaction, which involves computer-assisted bioinformatics technology such as molecular dynamics and the docking of designed peptides to a target protein. This approach was used by Spodzieja et al., who based the design of BTLA protein inhibitors on the crystal structure of the BTLA-HVEM complex. The results indicate that the HVEM (14–39) peptide (Table 1) is a potent inhibitor and competes with the HVEM protein to bind to the BTLA protein. This peptide interacts 2.5 times more strongly with the BTLA protein than HVEM, and in its sequence, the HVEM (14–39) peptide has four cysteines, forming two disulfide bridges, which allow for a structure that is similar to that of this peptide fragment in the HVEM protein [133,134]. A similar approach was used by the group of Thakkar et al., whose 17-residue cyclic peptide (P16, Table 1) binds to CTLA-4 with a strength of 31 µM and inhibits tumor growth in a co-culture of Lewis lung carcinoma. For efficient peptide design, the group combined Rosetta with molecular dynamics simulation and free-energy calculation techniques [135].

Another method that makes it possible to screen a million peptides without testing each one individually is a technology called phage display. This method uses special phage libraries in which peptides are presented on the surface of bacteriophage virions (most often the filamentary phage M13). The peptide phage display is used to select peptides that bind to the target protein. The genetic information on each variant of the peptide under study is contained in the genome of the phage used in the library, so simple DNA sequencing allows for its rapid identification [136]. Gurung et al. used this method to identify two peptides that bind to the PD-L1 protein. Both peptides (PD-L1Pep-1 and PD-L1Pep-2, Table 1) bind to PD-1 with strengths of 373 and 281 nM, respectively. Moreover, the researchers showed that these peptides accumulate at PD-L1, expressing the tumor location one hour after injection, and the antibody accumulates after 24 h [137]. Furthermore, to increase the strength of the PD-L1Pep1 interaction with the PD-L1 protein, 24 PD-L1Pep1 peptides were attached to the surface of the ferritin nanocage. Such treatment resulted in an approximately 12-fold (~30 nM) higher binding to the PD-L1 protein. In addition, doxorubicin was encapsulated into ferritin nanocage, resulting in enhanced anti-tumor activity compared to the anti-PD-L1 monoclonal antibody [138]. A peptide inhibitor (CLP002, Table 1) was also identified by phage display. CLP002 shows a high affinity for PD-L1, which is overexpressed in adenocarcinoma and prostate cancer tumors. Moreover, the peptide shows much better tumor penetration than the antibody [139]. A peptide targeting the CTLA-4 protein (LC4, Table 1) was also developed using peptide phage display. Although the peptide binds specifically to CTLA-4 and inhibits the formation of the CTLA-4-CD80 complex, Zhou et al. decided to modify the peptide to enhance anti-tumor activity. A tumor-targeting peptide (RGD) was used for the modification, and a short peptide with the sequence PLGLAG was used as a linker between LC4 and RGD. The modified peptide (LC4-PLG-RGD) exhibits enhanced anti-tumor activity than the LC4 fragment in vivo [140]. The phage display mostly uses standard amino acids; therefore, Zhou et al. applied mirror-image phage display bio-panning using the D-enantiomer of TIGIT protein. The selected ^D^TBP-3 peptide (Table 1) is composed of d-amino acids, making it resistant to proteolytic digestion. Researchers have shown that this peptide inhibits tumor growth in CD8^+^ cells [141].

One of the latest systems is random nonstandard peptide integrated discovery (RaPID), which is based on flexizyme technology and allows for the synthesis of peptides to generate macrocyclic peptide libraries containing >1 × 10^12^ unique peptides with modifications such as backbone N-methylation or macrocyclic backbones [142,143,144]. Macrocyclic peptide D4-2 (Table 1), consisting of 15 amino acid residues, was identified using the RaPID system. This peptide shows high affinity to mouse CD47 (*K_D_* = 8.22 nM) and inhibits the formation of the CD47-SIRPα complex in an allosteric manner [145]. A different approach was presented by Jeong et al., who used targeted evolution with yeast display to engineer a small protein based on the ectodomain of PD-1. The small PD-1 protein was more active than the anti-PD-L1 antibody in treating a mouse model of cancer [146]. In further studies, the β-hairpin peptide was isolated from engineered small PD-1 protein and conjugated with dendrimer to stabilize the peptide structure. Such a peptide–dendrimer conjugate (PDC) also significantly increased the PD-L1/PD-1 inhibitory effect compared to the unconjugated peptide [147].

In conclusion, peptides are an increasingly attractive group of compounds that can effectively block the function of negative immune checkpoints. This is supported by a recent publication in which Rodriguez et al. presented a macrocyclic peptide (pAC65), an inhibitor of the PD-L1/PD-1 complex formation, with a binding strength comparable to that of FDA-approved monoclonal antibodies, but with a favorable safety and pharmacokinetics profile. In addition, this peptide also interferes with the formation of another CD80-PD-L1 complex. This dual capability makes it a promising candidate for cancer immunotherapy [148]. Moreover, it is worth noting that the proper formulation of peptides with nanoparticles can largely eliminate their disadvantages, such as low stability and short half-life.

**Table 1:** Peptide sequences with the methods used to identify them and the determined binding constants to their targeted proteins.

Peptide Name	Peptide Sequence	Method of Identification	*K_D_* (μM)	Target
HVEM(14–39)	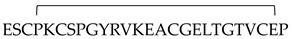	rational design [134]	0.102	BTLA
P16	cyc(EIDTVLTPTGWVAKRYS)	rational design [135]	31	CTLA-4
PD-L1Pep-1	CLQKTPKQC	phage display [137]	0.373	PD-L1
PD-L1Pep-2	CVRARTR	phage display [137]	0.281	PD-L1
CLP002	WHRSYYTWNLNT	phage display [139]	0.366	PD-L1
LC4	WGHSHFSHWKGR	phage display [140]	6.86	CTLA-4
^D^TBP-3	GGYTFHWHRLNP	phage display [141]	5.60	TIGIT
D4-2	RYSAVYSIHPSW	RaPID [145]	0.008	CD47

## 4. Nanomaterials for Cancer Immunotherapy

In recent years, the excellent therapeutic outcome of checkpoint blockade and chimeric antigen receptor (CAR) T cell therapy in cancer treatment brought promising hope to patients suffering from malignant tumors and inspired the following agent development and clinic trials [149,150]. However, the modest outcomes in subsequent clinic trials did not reach the expectation for effective tumor remission, while only part of the patients could finally reach a complete immune response [33,151,152,153]. In some cases, even though the therapeutic response could be observed at the initial stage, a developed resistance to blockade agents was a common issue in subsequent treatments, which will lead to a progression of the tumor and a poor prognosis [154,155]. For CAR T cell therapy, even though it displayed excellent therapeutic efficiency in malignant hematological tumors, such as leukemia and myeloma, the performance in solid tumor treatment was relatively mediocre [156,157,158]. At the same time, accompanied by the unsatisfactory treatment outcome, the safety concern in immunotherapy increases. Unlike traditional chemotherapy, in which the drug targets are solely expressed or highly up-regulated inside of the tumor, various immune cells are widely distributed all over the body, and could be mistakenly modulated by blockade agents, thus leading to inflammatory side effects (immune-related adverse event). Based on previous reports, immune-related adverse events are commonly observed in the gastrointestinal tract, endocrine glands, liver, skin, nervous system, and cardiopulmonary system, and the hematological system could also be involved [159,160]. All current issues highlight the urgent demand for novel methods in tumor immunotherapy, which could enhance therapeutic performance while minimizing the side effects.

Theoretically, dosage increases in the treatment could be an effective method to improve therapeutic efficiency; however, this strategy is challenging in immunotherapy since the targets for immune agents are also expressed in circulating immune cells and are thus widely distributed in different body systems. Therefore, the dosage needs to be carefully decided to reach a balance between obtaining maximal therapeutic outcomes and minimum immune-related adverse events. Even though the intratumor injection could avoid this perplexity, the complex operation and deficiency in metastasis treatment limited its application in the clinic. Therefore, optimizing the medicine delivery, which could improve drug uptake within tumors and limit its distribution in healthy tissue, could be a promising solution for addressing the dilemma that immunotherapy faces, and nanomedicine seems like a promising candidate for this method.

Nanomedicines are particle formulations of therapeutics encapsulated or conjugated by carrying materials (including polymers, lipids, or inorganic materials), with dimensions of 10–100 nm, and have been widely used in biological imaging and chemotherapy [161]. After intravenous administration, the distribution of nanomedicine into healthy tissue via the blood supply is restricted as the large size makes it difficult to penetrate the compact vascular wall [162]. Due to the structural abnormality in tumor vessels, which is featured by the deficiency of the basement membrane and fenestrated structure, nanomedicine could easily escape from the circulating system to the tumor [162]. At the same time, the abundant vessel distribution and deficiency of lymphatics further facilitate the accumulation of nanomedicine inside the tumor. Besides the passive tumor accumulation based on the above-mentioned mechanism (enhanced permeability and retention effect, EPR effect), the surface of nanomedicine could be further modified with various targeting moieties, including functional molecules, antibodies, and proteins, which could endow it with additional affinities towards the tumor, thus improving the medicine delivery efficiency and selectivity [161,163]. Moreover, owing to the large specific surface area, nanomaterials provide ideal adsorption sites for various plasma proteins, which could form a protein corona around the particle [163]. Since the interaction between the biological corona and nanoparticle is relatively weak, the innate biological properties of corona proteins will rarely be affected, which could still interact with multiple receptors (such as scavenger receptor and complement receptor) on phagocytes and increase the uptake by inducing phagocytosis [164]. Based on these features, nanomedicine could be an ideal delivery platform for immunotherapy agents, which could optimize the pharmacodynamics and pharmacokinetics, thus promoting their therapeutic efficiency and decreasing the side effects.

**Figure 7:**
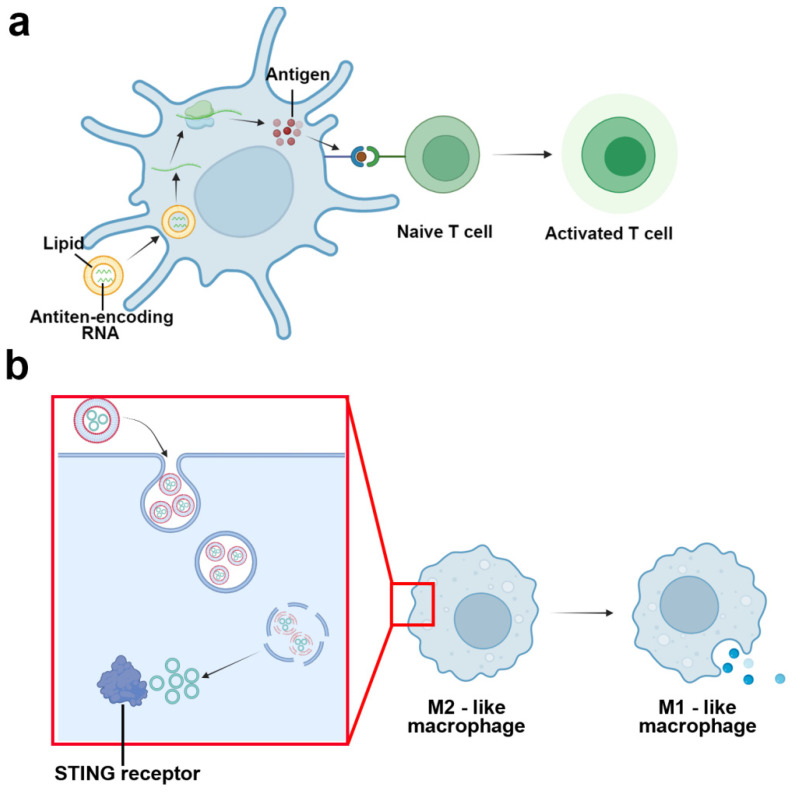
(**a**) Lipid-encapsulated antigen-encoding RNA is designed to enhance penetration efficiency and thus promote the tumor-specific antigen presentation efficiency, leading to the activation of T cells. (**b**) pH-sensitive liposome promotes the delivery of cGAMP into the cytoplasm via lysosome membrane disruption, which could facilitate the interaction with STING receptor and reprogram M2-like macrophage into M1-like phenotype.

In 2016, to promote dendritic cell uptake and avoid degradation from extracellular ribonucleases, Kranz et al. used lipids to encapsulate tumor-associated antigens encoding RNA and prepared them into RNA-lipoplex (Figure 7a) [165]. After intravenous administration, the selective distribution in immune organs could be accomplished by simply altering the net charge on the lipoplex without any requirement of surface modification. Compared with carrier-free RNA, RNA-lipoplex demonstrated enhanced RNA translation efficiency and improved therapeutic performance on multiple mice tumor models (B16-OVA lung metastasis model, B16F10-Luc tumor model, Luc-transduced, or wild-type CT26 tumor model, and advanced HPV16 E6- and E7-expressing TC-1 tumor model). In the following clinical trials (NCT02410733), for one patient with suspicious thoracic lymph node metastasis, after accepting quintic dosages of NY-ESO-1 encoding RNA-lipoplex, a rapid proliferation in antigen-specific CD8^+^ T cell could be observed with a regression in the metastatic site (validated by imaging examination) [165]. The phase I/II clinical trial for various antigen-encoding RNA-lipoplexes is still underway [166]. Cyclic GMP-AMP (cGAMP) is a cyclic nucleotide agonist for the STING pathway, which has been reported to be able to polarize M2-like TAMs to the M1-like phenotype. However, due to the endocytosis, soluble cGAMP will generally be trapped in lysosome after internalization and thus blocked from the interaction with the STING receptor in the cytoplasm, which would deteriorate the conversion efficiency [161]. In 2018, Cheng et al. tried to encapsulate cGAMP into liposomal nanoparticles to optimize the delivery efficiency (Figure 7b) [167]. As a result of lysosome acidification, the liposome could break the lysosome membrane after being phagocytized, thus releasing cGAMP into the cytoplasm. Compared with the soluble reagent, lipid-encapsulated cGAMP demonstrated enhanced antineoplastic ability, which could limit tumor growth and elevate survival in multiple tumor-bearing mice models. After treatment, the authors verified the phenotype program by monitoring the expression of M1/M2 relative genes on isolated CD11b+ TAMs, in which an up-regulated expression of M1-related genes (Il6, Nos2, and Tnaf) could be detected with a decrease in the expression of M2-related genes (Arg1 and Ym1), indicating the tilt of the M1/M2 ratio towards the pro-inflammation phenotype.

**Figure 8:**
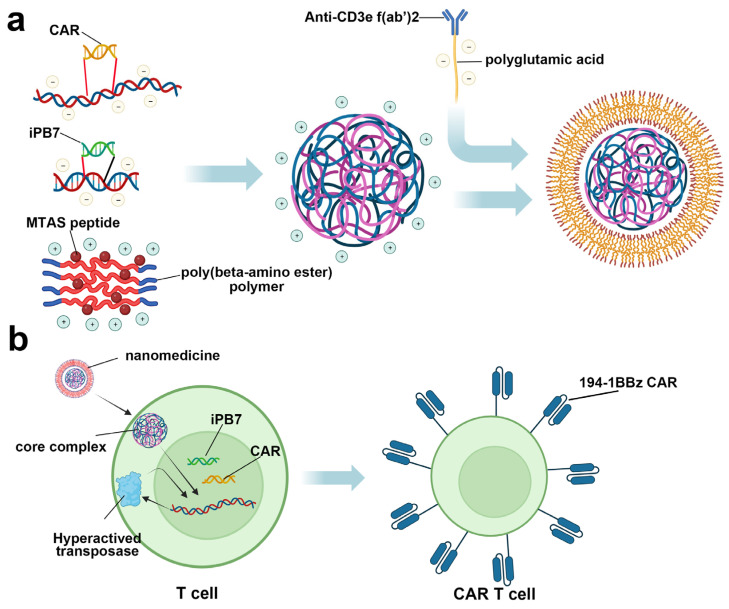
(**a**) Structure of the engineered nanomedicine for in vivo CAR gene insertion. The plasmid DNA with CAR- and iPB7-encoding sequence and poly(beta-amino ester) polymer containing MTAS peptide were mixed (1/30, *w*/*w*) to form the core complex, which was subsequently encapsulated by polyglutamic acid (modified with Anti-CD3e f(ab’)2) to provide the nanomedicine. iPB7: a hyperactive transposase to facilitate DNA integration. MTAS: microtubule-associated sequences used to facilitate the nucleus transport via the microtubule transport machinery. Anti-CD3e f(ab’)2: endorse the nanomedicine with T cell targeting ability. (**b**) Highly engineered nanomedicine for in vivo CAR gene insertion. After being phagocytized by lymphocytes caused by Anti-CD3e f(ab’)2, the biodegradable shell of nanomedicine will break the lysosome membrane and release the core complex into the cytoplasm, which could be easily imported to the nuclear by MTAS via the microtubule transport machinery. After being integrated into the host chromosome, the expression of 194-1BBz CAR could be accomplished. Moreover, the hyperactivated transposase encoded by the iPB7 sequence could further facilitate the above integration process.

Besides blockade agent delivery, nanomedicine was also reported to achieve in vivo CAR T therapy. In 2017, Smith et al. reported the development of a highly engineered nanoparticle for inserting a leukemia-targeted CAR gene into the T cell nuclei, which could edit circulating T cells into the antineoplastic phenotype in vivo and avoid complicated ex vivo operation (Figure 8) [168]. After intravenous administration on B cell acute lymphoblastic leukemia-bearing mice, CAR expression could be detected in 5.8 ± 0.9% CD3^+^ cells after 6 days post-treatment, and this number expanded to 7.1 ± 1.7% on day 12, with a significant tumor remission compared with the control group. More importantly, compared with conventional adoptive CAR T cell therapy, nanoparticle treatment displayed similar efficiency in both transgene and therapy, indicating the promising application prospect of this nanoparticle.

With the development of nanotechnology, the role of nanomedicine in immune modulation is not just limited to the delivery platform, as its application in immune activation has also been explored. Via an encapsulating photosensitizer, high-Z element, or paramagnetic material, nanomedicine could interact with external energy (such as light, radiation, and a magnetic field) and promote immune activity by inducing intense immunogenic death in tumors [161,169,170,171]. Additionally, some inorganic nanomaterials, such as ferumoxytol and Mg_2_Si nanoparticles, were reported to be able to program the tumor microenvironment into an activity statute, which provides a novel therapy strategy in immune therapy [172,173].

Similar to biological agents, small molecule inhibitors also face multiple challenges after systematic administration, such as low biostability, short circulating time, and limited tumor penetration ability. Encapsulating them into nanomedicine seems like a promising method to address these problems, just like the successful application in the above-mentioned biological agents. The water solubility of molecules is another challenge in medicine development. Generally, to pursue maximum therapeutic efficiency, molecular inhibitors usually possess a complicated structure with multiple hydrophobic groups, which will severely deteriorate the water solubility. And for the aromatic ring that is commonly used in medicines, even though it could increase the binding affinity of molecular inhibitors via forming a π-π interaction with some amino acid residues, molecule coagulation could also be caused in an aqueous solution for the same reason. For the above-mentioned reasons, even though some hydrophobic molecules might demonstrate excellent immune modulation ability in vitro, the possibility for any further development will still be eliminated. Fortunately, amphiphilic material-based nanomedicine seems like a perfect platform for these compounds, as it could provide a hydrophobic internal environment for encapsulated molecular inhibitors, while the surface is full of hydrophilic groups, which could keep its dispersity after injection. Since the development of molecular inhibitors is still in the preliminary stage, the application of small molecule-based nanomedicine in cancer immunotherapy is relatively less. However, they both displayed an improved therapeutic efficiency compared with free compounds in previously reported studies [174,175,176]. Both nanomolecules and CAR-T cell therapy have the potential to play crucial roles in the future of cancer treatment. The choice between them will likely depend on the specific characteristics of the cancer being treated, the goals of the therapy, and the ability to address and overcome their respective challenges. Combination approaches that leverage the strengths of both may be particularly promising.

## 5. Conclusions

Remarkable advancements in the treatment outcomes of cancer patients have been achieved through the implementation of immune checkpoint therapy. Currently, antibodies dominate checkpoint therapy, yet the low response rate and the occurrence of immune-related adverse events necessitate the development of more effective and safer treatment modalities. Small molecules and peptides offer the potential advantages of improved tumor penetration, minimal immunogenicity risk, amenability to pharmacokinetic optimization to avoid adverse events, oral bioavailability, and reduced off-target toxicities. Several approaches in the past decade have been implemented by researchers in the industry and academic groups that resulted in the discovery of first-in-class small molecule-based and peptide-based inhibitors of immune checkpoints with the potential for clinical translation. This review focuses on key strategies, including the HTS of random chemical libraries, virtual screening to develop focused chemical libraries, the development of degraders and covalent inhibitors, DEL screening, the computational design of peptides, and phage display to identify peptide-based inhibitors. In addition, we discuss the potential of nanomedicine to provide a potent solution for optimizing the pharmacokinetics and pharmacokinetics of small molecules and peptides in cancer immunotherapy, which could be a promising tool for promoting therapeutic efficiency in medicine development. In the coming decade and beyond, these strategies may increasingly improve our capacity to leverage the immune system against cancer, thereby maximizing the number of cancer patients benefiting from immunotherapy. The potential benefits of integrating small molecules and peptides into cancer immunotherapies could be significantly notable as the field advances toward synergistic combination therapies. These therapies are specifically designed to target multiple receptors with the goal of enhancing the overall response rates in cancer immunotherapy approaches.

## Data Availability

Not applicable.
